# Hormones Secretion and Rho GTPases in Neuroendocrine Tumors

**DOI:** 10.3390/cancers12071859

**Published:** 2020-07-10

**Authors:** Laura Streit, Laurent Brunaud, Nicolas Vitale, Stéphane Ory, Stéphane Gasman

**Affiliations:** 1 Institut des Neurosciences Cellulaires et Intégratives, Centre National de la Recherche Scientifique, Université de Strasbourg, F-67000 Strasbourg, France; laura.streit@inci-cnrs.unistra.fr (L.S.); vitalen@inci-cnrs.unistra.fr (N.V.); ory@inci-cnrs.unistra.fr (S.O.); 2 Département de Chirurgie Viscérale, Métabolique et Cancérologique (CVMC), Unité Médico-chirurgicale de Chirurgie Métabolique, Endocrinienne et Thyroïdienne (UMET), Unité Médico-chirurgicale de Chirurgie de L’obésité (UMCO), Université de Lorraine, CHRU NANCY, Hôpital Brabois Adultes, F-54511 Vandœuvre-lès-Nancy, France; l.brunaud@chru-nancy.fr

**Keywords:** neuroendocrine tumors, Rho GTPases, hormone secretion, vesicular trafficking, mutations, expression changes

## Abstract

Neuroendocrine tumors (NETs) belong to a heterogeneous group of neoplasms arising from hormone secreting cells. These tumors are often associated with a dysfunction of their secretory activity. Neuroendocrine secretion occurs through calcium-regulated exocytosis, a process that is tightly controlled by Rho GTPases family members. In this review, we compiled the numerous mutations and modification of expression levels of Rho GTPases or their regulators (Rho guanine nucleotide-exchange factors and Rho GTPase-activating proteins) that have been identified in NETs. We discussed how they might regulate neuroendocrine secretion.

## 1. Introduction

**Figure 1:**
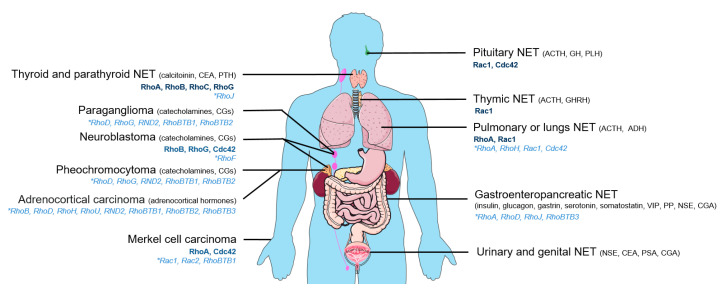
Main types of neuroendocrine tumors (NETs). Mutated Rho GTPases (light blue) or Rho GTPases for which expression level varies (dark blue) are indicated for each NET along with main secreted hormones. Hormone abbreviations: ACTH: adrenocorticotropic hormone, ADH: antidiuretic hormone, CEA: carcinoembryonic antigen, CGA: chromogranin A, CGs: chromogranins, GH: growth hormone, GHRH: growth hormone releasing-hormone, NSE: neuron specific enolase, PLH: prolactin luteinizing hormone, PP: pancreatic polypeptide, PSA: prostate specific antigen, PTH: parathyroid hormone, VIP: vasoactive intestinal peptide.

Neuroendocrine tumors (NETs) constitute a group of neoplasms that arise from cells secreting hormones, amines, or peptides. This family of tumors is highly heterogeneous in terms of morphology and function mainly because neuroendocrine cells are spread all over the body (Figure 1). The diffuse neuroendocrine system includes the neuroendocrine cells dispersed in various organs such as the thyroid (C cells), gastrointestinal tract, gallbladder, pancreas (islet cells), the respiratory tract, lungs, thymus, kidneys, liver, prostate, skin, cervix, ovaries, and the testicles. Few types of neuroendocrine cells actually constitute full organs such as the pituitary, paraganglia, parathyroid, and adrenal gland. From a cell biology perspective, the main common features of all these specialized cells are their ability to synthetize, stock in vesicles, and secrete, through calcium-regulated exocytosis, hormones, peptides, or amines. NETs are often associated with a deregulation of hormones secretion mainly leading to hypersecretion [1,2]. NETs with initial low secretory activity can evolve to high secreting lesions having a negative impact on prognosis simply by progressively becoming hormonally active [3,4,5,6,7,8]. Hence, cellular secretory activity appears to be a key controller of tumor behavior. However, how secretion becomes uncontrolled in NETs remains poorly understood.

Belonging to the Ras GTPase superfamily, the monomeric Rho (Ras homologous) GTPase family contains 20 highly conserved members divided into eight subfamilies (Rho, Rac, Cdc42, RhoD/F, Rnd, RhoU/V, RhoH, and RhoBTB) classified into two major groups. These include the canonical (Rho, Rac, Cdc42, and RhoD/F) and the atypical members (Rnd, RhoU/V, RhoH, and RhoBTB) [9,10]. In the last 10 years, many comprehensive reviews described Rho GTPases signaling as affecting a large array of cancer biology aspects through the control of important cellular processes including polarity, cell cycle progression, cytoskeleton organization, motility, and intracellular membrane trafficking [11,12,13,14,15,16,17,18,19]. Dysfunction of these crucial processes through aberrant Rho GTPases signaling can favor distinct steps of cancer progression from tumor initiation to tumor cell proliferation, invasion, and metastasis. Although such altered Rho GTPase signaling is linked to many types of cancer, to what extent pathways controlled by Rho GTPases are involved in NETs is still an open question. Seminal works from our team and others demonstrated that monomeric G proteins, including members of the Rho GTPases family, tightly control neuroendocrine secretion. However, the link between Rho GTPases and the NETs-associated deregulation of secretion remains largely unexplored. Here, we review the literature supporting the implication of Rho GTPases in NETs and discuss the possible links between Rho GTPases signaling and the regulation of neuroendocrine secretion.

## 2. Neuroendocrine Tumors and Rho GTPases

The first idea that usually comes to mind regarding the origin of tumors is genetic mutations. In contrast to other members of the Ras superfamily, Rho sub-family members were initially thought to be rarely mutated in cancer [20]. However, progress in advanced sequencing and better access to human samples allowed, in the last decade, the uncovering of many mutations in Rho GTPases (for review, see [12,17,20,21,22,23,24,25]). By searching the literature, we inventoried about 30 mutations or polymorphisms directly affecting Rho GTPases in NETs essentially in pheochromocytoma, paraganglioma, adrenocortical adenoma, small cell lung cancer, and Merkel cell carcinoma (Table 1, Figure 1, and Table S1). Surprisingly, besides the mutants Y42C-RhoA and P29S-Rac1, the impact of the other mutations on Rho activity and function remains unknown. P29S-Rac1 is a fast cycling mutant with spontaneous activation and therefore acts as a gain-of-function mutation [26,27]. The Y42C mutation reduces both intrinsic- and GAP-stimulated GTP hydrolysis of RhoA, thereby enhancing the active GTP-bound form [28]. On the contrary, Wang et al. proposed that the Y42C mutation decreased the level of the activated GTP-associated form of RhoA [29].

Beside mutations, variation in the expression levels of Rho GTPases has been described in many different types of tumors and at various stages of tumorigenesis (for previous key review articles, see [12,13,15,20,40,41,42,43]). However, only a few studies were performed in NETs, mainly in pituitary adenoma and neuroblastoma, as well as in tumors from the thyroid, parathyroid, and small cell lung. For instance, in pituitary adenoma, Rac1 overexpression and Cdc42 down-regulation may affect pathways controlling tumorigenesis such as mTOR- and Wnt-signalling pathways [44]. Arising from primitive cells of the sympathetic nervous systems, neuroblastoma is a common childhood extracranial solid tumor with neuroendocrine properties [45]. Although a large amount of molecular data were obtained from neuroblastoma, the situation appears complex as this tumor displays heterogeneous clinical behavior depending on multiple factors including tumor stage, patient age, and MYCN oncogene amplification. When these stratification parameters were used in these common childhood malignant tumors, different studies revealed modifications in the expression of proteins involved in Rho GTPases pathways (Cdc42, RhoG, RhoB, etc.) [46,47,48]. Most of these studies reported that deregulation of Rho GTPases pathways contributes to disease progression. Conversely, the most aggressive neuroblastoma presenting MYCN amplification also displayed down-regulation of Cdc42 expression through the control of N-myc, indicating that Rho GTPases overexpression is not always correlated with poor prognosis. Regarding thyroid or parathyroid tumors, elevated RhoA activity was correlated to the loss of proto-oncogene N-Ras and malignancy progression using the Rb1-deficient mice model of medullary thyroid (C-cell) adenomas [49]. A comprehensive proteomic study revealed differences in the expression levels of various Rho GTPases (mainly RhoA, B, C, and G) between medullary, anaplastic, and epithelium-derived differentiated thyroid cancers (for details see Supplementary Data in [50]). NETs represent around 25% of lung neoplasms with small cell lung cancer (SCLC), the most common and aggressive cancer [51]. In high-grade SCLC, RhoA is highly expressed [52,53], whereas Rac1 seems to be more abundantly expressed in low-grade pulmonary carcinoid tumors [54]. Finally, few studies reported the involvement of Rho GTPases in cervical, thymic, or skin (Merkel cells) tumors, most likely due to their low frequency. To the best of our knowledge, only one study performed on thymic carcinoid tissue reported Rac1 overexpression [55]. As Merkel cell carcinoma can be the consequence of oncogenic polyomavirus infection, the implication of RhoA and Cdc42 in the pathway by which virus small T antigen controls Merkel cells motility was proposed [56].

Overall, only few studies reported significant expression level modifications for Rho GTPases family members in NETs. It is, however, important to remember that Rho GTPases expression levels are not necessarily correlated with their activation levels. This balance has been largely overlooked.

## 3. Control of Rho Activity in NETs: Important Role of Rho GEFs and GAPs

The activity of most Rho GTPases is under the control of their regulators. Modulating the expression of guanine nucleotide-exchange factors (GEFs), which stimulate the exchange of GDP for GTP, as well as that of GTPases-activating proteins (GAPs) that catalyze GTP hydrolysis, are expected to alter Rho GTPases activity. For example, in pheochromocytoma, a NET arising from chromaffin cells of the adrenal medulla, we observed that the activity of Rac1 and Cdc42 was inhibited while their relative expression remained unchanged compared to non-tumor tissue [57]. In this study, we further showed that the inhibition of Rac1 and Cdc42 activities in human pheochromocytomas was directly correlated to reduced expression of the GEFs ARHGEF1 and FARP1, respectively [57,58]. In the very aggressive SDHB-pheochromocytoma, microRNAs controlling the Rho GAP ARHGAP18 expression are specifically overexpressed [59]. Expression level changes of Rho GEFs and Rho GAPs were reported in other different NETs from pancreas, lung, thyroid, prostate, and the pituitary gland (Table 2). For example, expression of Frabin (FGD4), a GEF specific for Cdc42, positively correlates with the aggressive phenotype of prostate cancer and the tumor grade of pancreatic neuroendocrine neoplasms, most likely by maintaining abnormal activation of Cdc42 [60,61]. Knock-down of FGD4 in PC-3 and LNCaP-104S prostate cell lines inhibited cell proliferation, cell cycle progression, and cell migration [60]. In pituitary adenoma, ARHGAP18 and ARHGEF17 are both upregulated, suggesting a modulation of the activity of the target Rho GTPases, most likely RhoA [44]. Variation in VAV isoforms (GEFs for Rho and Rac GTPases) expression levels was reported in small cell lung carcinoma [62,63].

By searching the literature, we found that Rho GEFs and Rho GAPs seem to be more affected than the Rho GTPases in another aspect. Strikingly, we found a tremendous amount of mutations and polymorphisms for Rho GEFs and GAPs in NETs, which seem to exceed those found for Rho GTPase genes by far (Table S2). However, most of the time, how these mutations and polymorphisms affect Rho GTPases activity, their consequences on Rho GTPases signaling, and their impact on tumorigenesis remain completely unknown and will require further investigations.

## 4. Rho GTPases and Hormones Secretion

One common aspect of NETs is the perturbation of hormone secretion, a cellular process regulated by Rho GTPases pathways [11,67,68,69,70,71]. Regulation of hormone secretion in neuroendocrine cells has been mainly studied in two in vitro models: the chromaffin cells from the adrenal medulla (primary culture of mice and bovine chromaffin cells or the rat pheochromocytoma cell line PC12) and the pancreatic beta cells (primary culture or the mouse insulinoma cell line MIN6) [72,73,74]. These two models are particularly relevant to further understanding the mechanisms of NET-associated hypersecretion. Human pheochromocytoma is characterized by catecholamine hypersecretion, leading to severe hypertension, cardiopathy, and high risk of stroke. In the pancreatic islet cells adenoma (insulinoma), insulin secretion is dysregulated with a persistent hypersecretion that may lead to severe hypoglycemia with associated-neuroglycopenic symptoms [75,76].

### 4.1. Control of Secretion through Actin Remodeling

In all kinds of tumors, Rho GTPases dysfunction is often linked to their role on actin cytoskeleton organization. Both in adrenal chromaffin and pancreatic beta cells, Rho GTPases were shown to play a key role in secretion by controlling actin remodeling. We demonstrated that the GTPases RhoA and Cdc42 play negative and positive roles on exocytosis, respectively, by differentially affecting actin organization [69,70,77]. Firstly, upon exocytosis, Cdc42 is activated at the plasma membrane of PC12 cells where RhoA is inhibited [67,78]. Following these early studies, RhoA was proposed to actively maintain the organization of the cortical actin network that controls granule positioning and likely limits their access to the plasma membrane in resting condition [77,79,80]. Consequently, inhibition of RhoA during exocytosis was postulated to be an essential step in promoting depolymerization of the cortical actin fence [78]. Conversely, once activated, Cdc42 recruits the neural Wiskott-Aldrich syndrome protein (N-WASP) at the exocytotic sites of the plasma membrane [78]. Subsequently, our observations allowed us to propose a model in which secretory granules tethering to the exocytotic sites allows the granule bound-actin-related protein-2/3 (Arp2/3) complex to interact with N-WASP and trigger actin nucleation and de novo polymerization of filaments that optimize the efficiency of the exocytotic process [69,78]. Accordingly, Rho GTPases-mediated actin organization tightly regulates insulin secretion in pancreatic cells islets according to a similar dual mechanism controlling actin polymerization: (i) F-actin network organized as a cortical negative barrier that restricts insulin-containing granule accumulation at the plasma membrane hence limiting basal insulin release and (ii) F-actin remodeling leading to a coordinated depolymerization of cortical actin and de novo polymerization or actin fiber assembly leading to positive effects on stimulus-induced insulin granule exocytosis [81]. Glucose-induced activation of Cdc42 was also shown to control insulin secretion in MIN6 pancreatic beta cells through the N-WASP-Arp2/3 or the PAK1-Rac1 signaling pathways, both leading to actin cytoskeleton remodeling [82,83,84].

How actin remodeling at the exocytotic sites controls hormone release is a key question that has attracted considerable attention, but that is not yet fully resolved. In BON cells, a pancreatic neuroendocrine cell line secreting serotonin, Cdc42, was shown to regulate fusion pore expansion through modulation of membrane tension [85]. As actin cytoskeleton is a known regulator of membrane tension, novel actin filaments generated by active Cdc42 may provide forces at the exocytotic sites to tense membrane and enhance fusion pore expansion and granule cargo release. The exact orientation of these novel actin filaments toward plasma and granule membranes has not been clearly established. Recently in bovine chromaffin cells, electron microscopy coupled to tomography revealed that actin bundles connected plasma and granule membranes of docked granules after exocytosis stimulation [78]. Accordingly, links between hormone secretion and coating of secretory granules with actin filaments or actin filaments anchoring secretory granules to the plasma membrane were described in chromaffin and insulinoma cells [86,87]. Usually, actin filaments need motors to provide forces to membranes. Rho GTPases were shown to regulate the activity of various myosins [88,89,90,91]. The involvement of myosin II and VI in endocrine secretion was described in adrenal chromaffin and PC12 cells, as well as in pancreatic BON and beta cells [85,92,93,94,95,96]. Together, these findings show that Rho GTPases may tightly regulate the polymerization status of F-actin in secreting cells, allowing for the close interplay of the negative control played by cortical actin and the positive action on exocytosis by de novo actin polymerization or bundling.

### 4.2. Control of Secretion through Lipids Action

Rho GTPases were also shown to control lipids metabolism pathways that are critical for neuroendocrine secretion [11,97]. In rat pheochromocytoma cells, we demonstrated that short interfering RNA (siRNA)-based knockdown of Rac1 inhibits hormone secretion by preventing the secretagogue-induced activation of phospholipase D1 (PLD1) [98]. PLD1 produces phosphatidic acid (PA), a coned-shape fusogenic lipid pivotal for efficient secretion in neuroendocrine cells including adrenal medulla and pancreatic islet cells [97,99,100,101]. Notably, PLD upregulation was shown to play various cellular and physiological roles in cancer [102,103]. Among the possible contributions of excessive PA synthesis in tumorigenesis, we mention the activation of the mTor pathway by PA that directly binds to mTor in a rapamycine-competitive manner and the increase in metalloprotease secretion triggered by PA [102,103].

A recent study highlighted the importance of the lipid transporter ABCA12 in insulin secretion. ABCA12 silencing in pancreatic β cells impaired secretory granule maturation and fusion, most likely through an altered cellular distribution of cholesterol between insulin granules and the plasma membrane lipid rafts required for secretion [104]. Remarkably, loss of ABCA12 expression also prevents Cdc42 activation and the associated actin remodeling [104].

Actin cytoskeleton remodeling and lipid organization are intimately linked during the process of hormone secretion [11]. For example, work from our laboratory demonstrated that formation of actin bundles connecting docked secretory granules to the plasma membrane contributes to the formation of GM1-enriched lipid microdomains at the exocytotic sites in chromaffin cells [86]. We showed that RhoA, which controls the organization of the cortical actin network at rest, can be recruited to the secretory granule membrane to regulate the phosphatidylinositol-4 kinase (PI 4-kinase) activity, hence modulating phosphatidylinositol 4-phosphate (PI4P) level [79]. How the level of PI4P on secretory granule membrane can impact secretion in currently unknown. One possible explanation is that PI4P is the precursor for phosphatidylinositol 4,5-bisphosphate (PIP2), a phosphoinositide that has been largely implicated in regulated secretion of hormones [105,106]. Coping with levels of phosphatidylinositol 4-phosphate 5-kinase (PI4P-5kinase), the enzyme that generates PIP2 from PI4P, dramatically affected exocytosis in chromaffin and beta pancreatic cells [105,106,107]. As RhoA activation diminished catecholamine secretion in chromaffin cells and since PIP2 controls many actin binding proteins, PIP2 might contribute to stabilizing secretory granules within the peripheral actin mesh.

### 4.3. Rho GEFs and Rho GAPs at the Commands

As mentioned above, one crucial checkpoint to insure physiological functioning of Rho GTPases is the tight regulation of their activation/inactivation cycle through the action of GEFs and GAPs proteins. Given uncovering the comprehensive mechanisms by which Rho GTPases regulate hormones secretion, we identified a set of different Rho regulators. In the chromaffin/PC12 cell models, stimulation of exocytosis triggers activation of Cdc42 and Rac1, associated with the inactivation of RhoA. We previously showed that the activation of Cdc42 is mediated by intersectin-1L, a member of the Dbl family of GEFs that also interacts with N-WASP and participates in actin organization [108,109,110]. In parallel, Rac1 is activated by β-PIX, a member of the Cool/Pix Rho GEFs family, which is recruited to the plasma membrane of stimulated-PC12 cells through its interaction with Scrib, the mammalian homologue of the Drosophila neoplasic tumor suppressor Scribble [98,111,112].

In pituitary and pancreatic cells, different GEFs have also been proposed to control hormone secretion. The transient activation of Rac1 required for glucose-induced insulin secretion was proposed to be under the control of VAV2, Tiam1, and Trio/Kalirin in pancreatic cells [113,114,115,116]. How these three GEFs coordinate spatially and temporally Rac activation needs further investigation. In the pituitary gland, the GEF trio has been also proposed to control hormone release [117].

Regarding RhoA, we proposed that Oligophrenin-1, a multi-domains GAP protein involved in various membrane trafficking events linked to synaptic functions (plasticity, post-synaptic receptor trafficking, and synaptic vesicle recycling [118,119,120,121,122]), might be responsible for the secretagogue-induced inactivation of RhoA [123]. Along the same line, inhibition of the RhoA/Rock pathway reduced neurotensin secretion in BON cells [124].

## 5. Conclusions

In comparison to other types of tumors, the role of Rho GTPases in NETs is not well documented. However, the high amount of genetic mutations and polymorphisms discovered in Rho GEFs and GAPs indicates that pathways controlled by Rho GTPases are likely affected in NETs. Today, a clear effort has to be directed toward understanding how mutations or variations in expression levels of Rho GTPases, GEFs, and GAPs identified in NETs favor tumorigenesis. Comparative genomic and proteomic analyses of human tumor samples remain among the most suitable general strategies to uncover new actors involved in Rho GTPases signaling.

Besides being a predictive factor for tumor occurrence or for its progression, whether Rho GTPases pathways could be used as therapeutic targets is clearly an aspect that needs to be developed in the near future. Several drugs directly targeting Rho GTPases have been recently designed and different strategies such as preventing Rho GEF interaction or inhibiting effectors have been proposed [125,126,127,128,129,130,131,132,133,134,135,136,137,138,139,140,141]. However, based on the complex involvement of Rho GTPases and their regulators in NETs hypersecretion, as reviewed here, the development of proper strategies to target each specific tumor will be critical and will require a perfect knowledge of the mechanisms leading to the deregulation of the Rho pathways, as well as their consequences on tumorigenesis.

## Figures and Tables

**Table 1:** Mutations and polymorphisms of Rho GTPases in NETs.

Gene/Tumor	ACC	GCC-AC	MCC (−)	NBL	PLCNEC	PNET	PPGL	PTC	SCLC	SINET	References
**RHOA**		Y42C							E40K		[30,31]
		D49H									
**RHOB**	D28H										[32]
**RHOD**	R174Q					R110Q	E149K				[32,33,34,35]
	A165T						A173P				
**RHOF**				K112 (fs)							[32]
**RHOG**							P139L				[32,34]
**RHOH**	A76E				P110A				S90 *		[30,32,36]
**RHOJ**								D8N		V25V	[32,37]
**RHOU**	S249Y										[32]
**RND2**	E168K						R169S				[32]
							R137T				
**RAC1**			P29S		P92P						[36,38]
**RAC2**			R68W								[38]
**CDC42**									K184N		[30]
**RHOBTB1**	R670 *		P366S				A575C (fs)				[32,38,39]
**RHOBTB2**	Q12H						D461G				[32,39]
**RHOBTB3**	R607H					S50T					[32,35]

Note: fs: frameshift, * STOP codon. Tumor abbreviations: ACC: adrenocortical carcinoma, GCC-AC: goblet cell carcinoid—adenocarcinoma, MCC (**−**) Merkel cell carcinoma-MCPyV negative, NBL: neuroblastoma, PLCNEC: pulmonary large-cell neuroendocrine carcinoma, PNET: pancreatic neuroendocrine tumor, PPGL: pheochromocytoma and paraganglioma, PTC: parathyroid carcinoma, SCLC: small cell lung cancer, SINET: small intestine neuroendocrine tumor.

**Table 2:** Expression level changes of Rho GEFs and Rho GAPs in NETs.

GEFs/GAPs	Protein/Gene	Expression Variation	Tumors with Expression Modifications	Preferential Targets of the GEFs and GAPs	References
GEFs	ARHGEF1	↘	PCC vs. non-tumor	RhoA	[57,58]
	ARHGEF10L	↗	NBL MYCN− vs. MYCN+ short survivors (gene)	RhoA, B, C	[48]
	ARHGEF17	↗	NFPA vs. non-tumor	RhoA	[44]
	FARP1	↘	PCC vs. non-tumor	Rac1	[57,58]
	FGD4	↗	PNET grade 2, 3 vs. 1	Cdc42	[61]
		↗	NEPC (gene)		[60]
	RCC2	↗	SCLC	Rac1	[54]
	VAV1	↘	uSCLC	RhoA, Rac1	[62]
		↗	SCLC cell lines vs. non-SCLC cell lines		[63]
	VAV3	↘	CRPC-NEPC	RhoA, RhoG, Rac1	[64]
GAPs	ARHGAP6	↗	PHPT vs. non-tumor	RhoA	[65]
		↘	NEPC (gene)		[66]
	ARHGAP11A	↗	NEPC (gene)	RhoA	[66]
	ARHGAP11B	↗	NEPC (gene)	RhoA, Cdc42	[66]
	ARHGAP18	↗	NFPA vs. non-tumor	RhoA, B, C	[44]
		↗	PCC (miRNA)		[59]

Up (↗) or down (↘) expression variations concerning proteins except when indicated (gene, miRNA). When available, the control (vs.) is indicated, as well as the main targeted-Rho GTPases. Tumor abbreviations: CRPC-NEPC: castrate-resistant prostate cancer-neuroendocrine prostate cancer, NBL: neuroblastoma, NEPC: neuroendocrine prostate cancer, NFPA: non-functional pituitary adenoma, PCC: pheochromocytoma, PHPT: primary hyperparathyroidism parathyroid adenoma, PNET: pancreatic neuroendocrine tumor, SCLC: small cell lung carcinoma, uSCLC: undifferentiated small cell lung carcinoma.

## Supplementary Materials

The following are available online at https://www.mdpi.com/2072-6694/12/7/1859/s1, Table S1: Mutations and polymorphisms of Rho GTPases in NETs, Table S2: Mutations and polymorphisms of Rho GTPases GEFs and GAPs in NETs.
